# Using Chemical Approaches to Understand RNA Structure and Function in Biology

**DOI:** 10.1155/2012/972575

**Published:** 2012-02-16

**Authors:** Dmitry A. Stetsenko, Arthur van Aerschot

**Affiliations:** ^1^Department of Bioengineering, Imperial College London, South Kensington Campus, London SW7 2AZ, UK; ^2^Department of Pharmaceutical Sciences, Katholieke Universiteit Leuven, Minderbroedersstraat 10, 3000 Leuven, Belgium

Interest of the research community in the aspects of chemical biology of RNA has increased vastly over the last twenty years, primarily due to the discovery of RNAi, our deepened understanding of the role of miRNA in the subtle regulation of vital cellular processes, and the realization of the fact that the RNA world—the realm where RNA plays the key parties as a self-replicating molecule and a universal catalyst—is still pretty much with us today as the ribosomal RNA performs its catalytic solo in the formation of the peptide bond in the ribosome. To answer the needs of Biology, Chemistry had, in turn, multiplied and perfected its approaches to study the molecular mechanisms underlying RNA functions in living systems. So, the idea behind this special issue is to show our readership a screenshot of what could be, and has been, achieved recently by applying chemical methods to solve the problems of RNA biology. Ten articles have been carefully selected out of the bunch of those submitted to provide, we believe, a balanced view of different facets of RNA structure and function and, also, of the array of chemical tools to enable us to peek into them.

The issue is a mix of reviews and research papers and thus falls into two parts. We have purposefully incorporated a number of authoritative overviews to paint a wider background of RNA functioning in the first half. It starts with a general treatise on chemical approaches to study RNA structure-function relationships in the postgenomic era by T. S. Ro-Choi and Y. C. Choi. Dependence of RNA-protein interaction on RNA conformation is highlighted by a medicinally important example of a noncoding RNA BC1 and the Fragile X Mental Retardation protein in another paper by X. Yan and R. B. Denman. The one that follows by D. Kurita et al. amply shows how chemical probing has provided insights into the mechanisms of *trans* translation and the role played by transfer-messenger RNA (tmRNA). A Herculean task of the identification of minor nucleotides in RNA and the use of specific chemical reagents for its accomplishment are reviewed in the contribution from a group of French researchers led by Professor Y. Motorin. Taking snapshots of RNA in live cells by means of molecular beacons technology is the subject of a review of R. Monroy-Contreras and L. Vaca.

Then the issue goes into details of the interplay between a single chemical, resveratrol, and miRNAs during inflammatory processes and cancer, reviewed by E. Tili and J.-J. Michaille. The miRNA theme continues in the review by A. Serva et al. of the application of high-throughput functional analysis for elucidation of the multiple biological roles of miRNAs. Here we cross the boundary into the second half of our special issue composed from research papers, and the one that comes next by K. Terazawa et al. opens the RNA interference section with synthetic short hairpin RNAs (shRNAs) as potent RNAi inducers. Another article, commissioned by researchers from the Iberian Peninsula R. Eritja and coworkers, deals with branched RNA architectures for RNAi.

Finally, a paper of the issue done by M. A. Zenkova et al. focuses on an applied aspect of RNA technology, namely, the development of sequence-specific artificial ribonucleases incorporating multiple imidazole residues that perform similar function to their counterparts in natural RNA-cleaving enzymes. 

In conclusion, the issue attempts to present a selection of reviews and research papers, which are designed to illustrate how by using various implements and gadgets from the ever-growing arsenal of Chemistry one can hopefully gain a better understanding of what is happening when RNA molecules interact with their manifold targets and perform their plethora of functions. It must be added that this is by no means a comprehensive picture but rather a momentary screenshot of a dynamic process of harnessing the power of Chemistry to serve the ends of Biology. Sapienti sat. 

*Dmitry A. Stetsenko* *Dmitry A. Stetsenko* *Arthur van Aerschot* *Arthur van Aerschot*

